# Differences in Experiencing Well-Being in Youth Choir Singers Regarding (In)Formal Participation

**DOI:** 10.3390/bs15101337

**Published:** 2025-09-29

**Authors:** Jovana Blagojević, Katarina Habe, Boštjan Bajec

**Affiliations:** 1 Academy of Music, University of Ljubljana, 1000 Ljubljana, Slovenia; 2 Faculty of Arts, University of Ljubljana, 1000 Ljubljana, Slovenia; bostjan.bajec@ff.uni-lj.si

**Keywords:** choir singing, extracurricular choir, school choir, youth well-being

## Abstract

Choral singing is a widely practiced form of group music-making that has been associated with various positive well-being outcomes. While existing research highlights its benefits for adults and clinical populations, how choir participation affects healthy youth is explored to a lesser extent. This quantitative study examined differences in well-being between informal (extracurricular) and formal (school) youth choir singers in Slovenia and Serbia. A total of 274 choir members aged 15–24 completed an online questionnaire, including extracurricular (n = 77) and school (n = 197) singers. Standardized instruments tested general well-being (WHO-5), life satisfaction (SWLS), positive mental health (MHC-SF), emotional states in choir and in life (PANAS), social support (SPS-10), and perceived benefits from choral singing (CAPBES). Findings revealed that extracurricular choir singers reported significantly higher social connectedness and overall well-being subscales related to perceived benefits from choir participation, and passion in life, compared with school choir singers. School members had higher scores in subscale self-disgust within the negative affect in life scale. Positive and negative emotions within choir were similar for both groups. No significant differences were observed in life satisfaction, mental health, and social support in life. These results indicate that voluntary, informal (extracurricular) choir participation is associated with enhanced well-being, stronger social benefits, and higher positive emotions in life, compared with formal (school) choir participation. Integrating more voluntary choir opportunities into educational and community programs may increase inclusion and provide positive youth development.

## 1. Introduction

According to the World Health Organization (2024), youth around the world are facing increasing mental health challenges, with rising rates of anxiety, depression, loneliness, and social disconnection. These issues are particularly prominent during adolescence, a transitional period to adulthood, marked by significant emotional, social, and psychological development (Arnett, 2013). Because of this, the need for accessible, meaningful activities that promote youth well-being has become a priority in public health and education.

Choral singing is one of the most common, widespread, and culturally embedded musical activity across the world and is associated with multidimensional benefits for well-being. Clinical populations have been widely explored regarding beneficial effects from choir participation, including individuals diagnosed with dementia (Lee et al., 2022), chronic diseases, cancer (Fancourt et al., 2019), and physical/intellectual disabilities (Fancourt et al., 2022; Viola et al., 2024).

The benefits from choral singing for (older) adults have also been frequently explored in previous studies. Choir participation has demonstrated many social benefits for adults, such as an increased sense of belonging (Fernández-Herranz et al., 2021), social inclusion (Livesey et al., 2012), and social connection (Glew et al., 2020; Livesey et al., 2012). Unlike solo performances, choir participation requires collaboration, mutual listening, synchrony, shared responsibility, and connection among members. Choir has shown its power as a mood-regulating tool, helping in reducing stress (Good & Russo, 2021) and stimulating joy and happiness (Damsgaard & Brinkmann, 2022; Juan-Morera et al., 2024; Linnemann et al., 2017). It has demonstrated benefits for expressing emotions more freely (Eiksund, 2022). Being a part of a choir has been associated with improved mental health through nurturing self-esteem, enhancing satisfaction with life, providing a sense of purpose, and providing meaningful personal growth (Clift et al., 2010; Gul, 2018; Moss et al., 2018).

Despite this, relatively few studies have focused specifically on healthy youth (Table 1), including adolescents, aged 15–19 (Glew et al., 2020; Koulouriotou & Papakitsos, 2023; Parker, 2014; Petrović, 2023; Stojanović & Milošević, 2019), and emerging adults, aged 20–24 (Acquah, 2016; Barrett & Vermeulen, 2019; Linnemann et al., 2017). Research indicates that choral singing has a positive impact on multiple aspects of youth well-being. However, the majority of these studies did not specifically focus on either school-based (formal) or extracurricular/community (informal) choir settings and often did not specify the type of choir participation involved. In this review, choir settings were classified based on contextual information provided in each study.

### 1.1. School-Based (Formal) Participation

Studies focusing on school/university choirs and formal participation highlight a structured environment where choral singing contributes to a sense of responsibility, discipline, and motivation for academic achievements (Stojanović & Milošević, 2019). In school (formal) context, participation is often linked to educational outcomes. The study by Koulouriotou and Papakitsos (2023) demonstrated students (choristers) reported feeling calm, happy, and relieved during and after rehearsals. Acquah (2016) found university choir singers experienced increased confidence, focus, and a stronger sense of accomplishment, in mandatory choir participation. Additionally, the study demonstrated choir reduced stress and anxiety, improved overall mood of choristers, provided a sense of belonging, and provided more social connections. Studies (Acquah, 2016; Koulouriotou & Papakitsos, 2023) have shown choir participation improved self-esteem, motivation, and social skills and provided physical benefits (better posture and breathing) for choir singers. However, formal settings also posed some challenges, including too many rehearsals, peer judgment, cliques, and sometimes even mockery (Koulouriotou & Papakitsos, 2023; Parker, 2014).

### 1.2. Extracurricular (Informal) Choir Participation

Extracurricular (informal) or community choir settings involve a more voluntary and self-motivated nature of engagement. Linnemann et al. (2017) highlighted emotional benefits from participation in a university choir, where choristers reported better mood and relaxation following each rehearsal, as well as fewer negative emotions, like tension or stress. The study also found that while participants enjoyed the social aspects of choir, it was not a primary motivation for their involvement. Instead, their passion for music played a more significant role, especially since they had enough social support outside of the choir settings. Barrett and Vermeulen (2019) examined a multicultural university choir in South Africa, with voluntary participation. The study highlighted four main values of choir participation: musical, personal, resilience, and social. Choristers were primarily motivated by a deep love for music and described choir as a source of emotional relief from academic stress, personal fulfilment, and physical health. The study emphasized the development of life skills such as discipline and time management. A sense of belonging, cultural connection and interaction with other choir members, social inclusion, support, and integration were highlighted as major social benefits. Petrović (2023) examined the impact of participating in extracurricular choirs on youth well-being. Children, parents, and conductors reported benefits in social (building friendships, teamwork, a sense of belonging, and reduced loneliness), emotional (mood regulation, relaxation, and less stress), and psychological (reduced shyness, improved concentration, creativity, self-confidence, and discipline) aspects. Musical development was also evident, including better singing skills, music literacy, and performance abilities. While some challenges were noted in these studies, such as performance anxiety prior to concerts (Linnemann et al., 2017), language barriers, being scared of making mistakes while singing, high rehearsal frequency (Barrett & Vermeulen, 2019), performance pressures, stage fright, and time commitment (Petrović, 2023), the overall findings point to the choir’s significant role in fostering personal growth, resilience, mood regulation, and a strong sense of community among diverse participants.

### 1.3. Formal and Informal Choir Participation

Several studies have examined youth choir participation across both formal and informal settings, without explicitly analyzing the differences between these settings. The literature review by Glew et al. (2020) primarily focused on school-based choir studies, with 9 out of 13 conducted in educational settings, while the remaining 4 took place in diverse community contexts, such as camps or churches. The study found choir singing in school settings enhances social inclusion, connectedness, and personal growth (confidence and more positive self-perception). Studies regarding community or church choirs also reported positive psychological outcomes including greater cooperation among youth, self-esteem, and emotional regulation. The study by Parker (2014) emphasized the role of school choirs in shaping social identity, such as having future music career goals, enhancing pride because of representing school and receiving external validation and praises (awards), reputation, and leadership development.

Overall, previous studies have mainly focused on either school or extracurricular choirs and often small, culturally specific samples, which limits the generalizability of the findings. The purpose of this study is to examine a larger and more diverse sample from two European countries to explore differences in perceived well-being, positive mental health, social support, and benefits of choral activity in choir between school choirs singers and extracurricular choir singers.

**Hypothesis** **1.** *There are differences in perceived (1) well-being (emotional, social, and psychological), (2) positive mental health, (3) social support, and (4) benefits of choral activity in choir singers between singers in school choir and singers in choirs as an extracurricular activity.* 

## 2. Materials and Methods

### 2.1. Procedure

This cross-sectional quantitative study is a part of a larger body of research conducted within the framework of a doctoral dissertation, which explored well-being between formal (school) and informal (extracurricular) youth choir singers. A structured online questionnaire was designed using validated scales and additional questions for the purposes of the study, to assess different aspects of well-being associated with choir participation.

The questionnaire was created in both Slovenian and Serbian languages through the 1ka online survey platform https://www.1ka.si/d/en (accessed on the 9 September 2024). The data collection was conducted between September and October 2024 and distributed through multiple channels to reach a larger number of potential participants, including emails to choir conductors, choral associations, school directors, Facebook groups for choir communities, and popular messaging applications such as WhatsApp and Viber.

Prior to participation, participants were presented with an informed consent statement, detailing the purpose of the study, the estimated time for completion, the voluntary nature of participation, data confidentiality, and their right to withdraw at any time before submission. At the end of the questionnaire, participants provided an anonymous signature (such as initials or a symbol) to confirm voluntary participation while maintaining confidentiality.

### 2.2. Participants

For the purposes of this study, we included 274 youth choir singers, aged from 15 to 24 years, from Slovenia and Serbia. The sample consisted of 197 youth singers from (school) choir and 77 attending informal (extracurricular) choir, reflecting the predominant role of schools as main organizers of choral singing activities. The majority of participants were female participants, representing 81.4% of the sample, while 18.2% were male singers, which is consistent with typical sex distributions in choirs globally. Additionally, one (0.4%) participant identified as other. The age distribution showed that 72.3% of participants were adolescents (aged 15–19), while 27.7% were emerging adults (aged 20–24). Among adolescents, 53% were involved in school choirs and 15% in extracurricular choirs. In contrast, among emerging adults, 14.6% participated in school choirs, whereas 13.1% participated in extracurricular choirs. Adolescents were predominantly represented in school choirs, while emerging adults were more evenly split between school and extracurricular choirs. This overlap Regarding the country, 65.3% of participants were from Slovenia and 34.7% from Serbia. When analyzing choir type (female/male/mixed), 53.6% participated in mixed choirs, 39.4% in female choirs, and only 6.9% in male choirs, again highlighting the sex imbalance in choral singing (Table 2).

When examining the distribution of formal (school) and informal (extracurricular) choir participation across age groups, it is evident that among adolescents (15–19), the majority are engaged in school choirs (79.7%), with a smaller proportion participating in extracurricular choirs (20.8%). In contrast, emerging adults (20–24) demonstrate a more balanced distribution between school/university choirs (52.6%) and extracurricular choirs (46.8%) (Table 3). These figures, presented within each age group, suggest that older singers increasingly participate in choirs voluntary, whereas adolescents are more often engaged in school choirs due to structured educational requirements.

### 2.3. Questionnaire Structure

The online questionnaire, created in Slovenian and Serbian versions, consisted of a multiple validated psychometric instruments to assess different dimensions of well-being, positive mental health, social support, and perceived benefits related to choral singing. The questionnaire combined standardized instruments and additional choir-related questions developed by the researchers.

The first section of the questionnaire collected basic demographic data, including age, sex, country, type of choir (female/male/mixed), choir settings (school/extracurricular), and frequency of choir rehearsals per week. The second section explored various aspects of well-being (psychological, social, and emotional), social support, positive mental health, and perceived benefits from choral singing.

#### 2.3.1. Instruments

##### WHO-5 Well-Being Index

The WHO-5 questionnaire (Topp et al., 2015) was used to measure participants’ subjective psychological well-being in the last two weeks, consisting of five positively phrased items (e.g., “I have felt cheerful and in good spirits”), each rated on a 6-point Likert scale ranging from 0 (“At no time”) to 5 (“All of the time”) (Bech, 2004).

##### Satisfaction with Life Scale (SWLS)

The SWLS was used to measure individuals’ cognitive judgements of their subjective life satisfaction (Diener et al., 1985), consisting of five items (e.g., “In most ways my life is close to my ideal”), each rated on a 7-point Likert scale, ranging from 1 (“Strongly disagree”) to 7 (“Strongly agree”). The total score ranges from 5 to 35, with higher scores reflecting greater overall life satisfaction.

##### Mental Health Continuum-Short Form (MHC-SF)

The MHC-SF questionnaire (Keyes, 2009) was used to assess participants’ overall positive mental health. This instrument has demonstrated strong psychometric properties, and it is appropriate for adults, as well as adolescents (Lamers et al., 2011). It has been validated in the Canadian sample (Orpana et al., 2017). The MHC-SF for adolescents consists of 14 items, divided into three subscales: emotional well-being (3 items), social well-being (5 items), and psychological well-being (6 items). Responses were recorded on a 6-point Likert scale, ranging from 0 (“Never”) to 5 (“Every day”). The final results can be categorized into one of three mental health states: flourishing (high levels of well-being), languishing (low levels of well-being), and moderate (neither flourishing nor languishing). It is important to note that only in this particular scale, lower scores indicate higher mental health and flourishing.

##### Social Provision Scale (SPS-10)

The SPS-10 is an abbreviated version of the original Social Provision Scale developed by Cutrona and Russell (1987) and was validated across diverse populations (Caron, 2013; Sischka et al., 2024). It is used to measure individuals’ perceived social support. The short version includes five subscales: emotional support or attachment, social integration, reassurance of worth, tangible help, and orientation. Each item (10) is rated on a 4-point Likert scale from 1 (“Strongly disagree”) to 4 (“Strongly agree”). A specific characteristic of this short form scale is the absence of the neutral response option, which encourages participants to take a clear stance on each statement. All items are positively framed, and higher scores indicate higher levels of perceived social support.

##### Positive and Negative Affect Schedule (PANAS)

The PANAS questionnaire, developed by Watson et al. (1988), was used to assess participants’ emotional well-being in two distinct contexts: everyday life and during choir singing. The original PANAS instrument consists of 20 items, evenly divided into two broad dimensions: positive affect (PA) and negative affect (NA).

##### Choral Activity Perceived Benefits Scale (CAPBES)

The CAPBES questionnaire (Fernández-Herranz et al., 2021) is a self-reported measure designed to assess perceived benefits from choral singing. The scale includes 22 items, distributed across five subscales: ability, satisfaction, optimism, group engagement, and belonging. Responses are rated on a 5-point Likert scale, ranging from 1 (“Strongly disagree”) to 5 (“Strongly agree”), with higher scores indicating more positive experiences within the choir. A previous study by Hylton (1981) provided initial items in CAPBES, which highlighted psychological, communicative, integrative, musical-artistic, spiritual, and achievement dimensions. Subsequently, the CAPBES instrument was analyzed for its reliability and validity, by 35 choir directors, conducted on a sample of 1513 participants. That process further refined the scale and eliminated redundant items (65), resulting in 22 items in the final version of scale.

In addition to the standardized instruments previously described, we incorporated supplementary questions specifically developed for the purposes of this study to capture more nuanced aspects of choir-related well-being.

We included questions regarding the specific elements of the choir experience participants believed most influenced their well-being, including realizing one’s vocal potential, social interaction and bonding with other choir members, public performances and concerts, engaging with diverse repertoire, and attending choir competitions. Participants rated on a 5-point Likert scale, ranging from 1 (“Not at all”) to 5 (“Very much”), the extent to which each factor contributes to their psychological well-being. It allowed for a more detailed exploration of the specific elements of choral activity that may enhance youth well-being.

### 2.4. Data Analysis

Data analysis was conducted using IBM SPSS Statistics (Version 27) and Jamovi (Version 2.3) (The Jamovi Project, 2022) software. The initial stage involved data cleaning, where incomplete, inconsistent, or ineligible responses that did not fit into the criteria (age range, incomplete questionnaire, or omitted required signature) were removed to ensure integrity and quality of the dataset. Descriptive statistics (frequencies, means, and standard deviations) were then calculated to provide an overview of the sample characteristics (age, sex, choir type, choir settings, and rehearsal frequency).

#### 2.4.1. Validity and Reliability

To ensure validity and reliability of the instruments for the target population, measures were chosen based on a review of the music-related literature and prior validations. Before performing the main analysis, each instrument was further evaluated through exploratory factor analysis (EFA), confirmatory factor analysis (CFA), and structural equation modeling (SEM) using Jamovi (Version 2.3) (The Jamovi Project, 2022) software. This allowed for the refinement of measurement models and improved construct validity.

Several instruments were restructured based on the factor analysis. While the original scales were preserved, items were regrouped into revised latent factors better suited to experiences of Slovenian and Serbian youth choir singers. Model fit was assessed using standard indices, including the comparative fit index (CFI), the Tucker–Lewis index (TLI), and root mean square error of approximation (RMSEA). Model fit was considered acceptable based on established criteria: CFI and TLI values greater than 0.90 indicate a good fit (Bentler, 1990; Bentler & Bonett, 1980), while RMSEA values less than or equal to 0.80 are considered as acceptable (Browne & Cudeck, 1993). Reliability for each instrument was evaluated using Cronbach’s alpha (α).

#### 2.4.2. Factor Refinement

Reliability across the scales (Table 4) ranged from good and acceptable to excellent (α = 0.678–0.926). Only PAS in life fell slightly below the conventional 0.7 threshold. After the factor analysis, PAS in life was revised and categorized into three factors including engagement (interested, proud, alert, determined, and active), passion (enthusiastic, inspired, and attentive), and energy (excited and strong). The internal consistency was modest (α = 0.678) even after the refinement, and the model fit was slightly under the acceptable value (CFI = 0.839, TLI = 0.773). The negative affect scale (NAS) in life was also adopted to a three-factor structure including fear (distressed, scared, nervous, jittery, or afraid), self-disgust (upset, guilty, or ashamed), and hostility (hostile or irritable). This version showed improved reliability (α = 0.782); however, the model fit remained poor (CFI = 0.772, TLI = 0.680). The lower reliability and model fit for the PAS in life and NAS in life may be due to nature of assessing the emotional states in general, without specific context, making it difficult for participants to interpret and rate their emotional states consistently.

Instruments that were related to choir experiences (PAS in choir, NAS in choir, and CAPBES) demonstrated high reliability after factor refinements, demonstrating improved psychometric quality. The PAS in choir was revised into two factors including motivation (alert, inspired, determined, attentive, or active) and excitement (interested, excited, strong, enthusiastic, or proud), showing strong internal consistency (α = 0.876) and acceptable model fit (CFI = 0.959, TLI = 0.946). The NAS in choir was restructured into two subscales—tension (upset, guilty, scared, nervous, or jittery) and agitation (hostile or irritable), demonstrating good reliability (α = 0.847) and acceptable model fit (CFI = 0.910, TLI = 0.842), with one item (scared) excluded during refinement. The CAPBES instrument was reorganized into five aspects: ability, accomplishment, well-being, social connectedness, and optimism, demonstrating strong internal consistency (α = 0.926) and acceptable model fit (CFI = 0.922, TLI = 0.909). Overall, participants responded more consistently to choir-related measures than to general emotional states.

The MHC-SF, originally designed to measure three dimensions of mental health, did not fit a three-factor model in our sample. It was structured into a single-factor scale representing overall positive mental health, which improved coherence and demonstrated excellent reliability (α = 0.908) with acceptable indices (CFI = 0.865, TLI = 0.841). The SPS-10 was categorized into two distinct factors including social connectedness and social support. This structure showed high internal consistency (α = 0.901) and excellent model fit (CFI = 932, TLI = 0.908).

## 3. Results

### 3.1. Rehearsal Frequencies

The participants from both school and extracurricular choir group reported the average number of choir rehearsals attended weekly (Table 5). Responses ranged from one to four times per week, presenting the diversity of engagement intensity among the sample. The most common rehearsal frequency overall was twice per week (44.9%), followed by once per week (37.1%). A smaller proportion (10.7%) attended rehearsals three times per week. Very few participants attended rehearsals four times per week (1.1%), while one participant reported attending three to four times per week (0.4%). Last, 2.9% of participants reported rehearsal attendance one to two and two to three times per week. However, the frequency differed notably between the groups.

Among school choir members, the majority (55.3%) attended rehearsals twice a week, while 23.3% rehearsed once a week, and 13.7% rehearsed three times per week. Smaller proportions attended rehearsals more intensively such four times per week (1.5%), indicating relatively frequent rehearsal schedule within formal (school) choirs. The majority of extracurricular choir members (71.4%) reported rehearsing once per week, with only 16.9% attending bi-weekly rehearsals. Very few rehearsed more than twice per week (2.6%), suggesting a less intensive and more flexible schedule in informal (extracurricular) choirs. However, the unequal number of participants in each group (197 in school and 77 in extracurricular choir) may have influenced the percentage distributions. Overall, the data indicate that school choirs tend to involve more frequent and structured rehearsals, while extracurricular choirs are more likely to offer lower-intensity schedules.

### 3.2. Well-Being

To test the proposed hypothesis regarding differences in well-being, mental health, social support, and perceived benefits from choral singing between groups (school and extracurricular choir singers), descriptive statistics (Table 6) and independent sample t-tests (Table 7) were performed across all well-being measures (WHO-5, SWLS, PANAS in life and choir, MHC-SF, SPS-10, and CAPBES).

#### 3.2.1. General Well-Being and Life Satisfaction

No significant differences were observed in general well-being between the school (formal) and extracurricular (informal) choir singers. While extracurricular choir participants scored higher on the WHO-5 (*M* = 2.90, *SD* = 0.85) compared with school choir participants (*M* = 2.69, *SD* = 0.88), the difference did not reach statistical significance (*t* = −1.815, *p* = 0.072). For the life satisfaction (SWLS), also no significant differences were observed between the groups (*t* = 0.488, *p* = 0.626).

#### 3.2.2. Emotional Well-Being

##### General Life Context

For the PAS in life, significant differences were observed between the groups in the passion subscale (*t* = −2.310, *p* = 0.022). Extracurricular choir singers (*M* = 3.60, *SD* = 0.85) scored higher than school choir singers (*M* = 3.33, *SD* = 0.88), reflecting greater enthusiasm and inspiration in life. No significant differences emerged in energy (*t* = 0.148, *p* = 0.883) and engagement (*t* = −0.231, *p* = 0.817) subscales. Regarding negative emotions in life, the only significant difference was found in self-disgust subscale (*t* = 2.292, *p* = 0.023), with school choir singers (*M* = 3.06, *SD* = 0.727) reporting higher levels than extracurricular choir singers (*M* = 2.84, *SD* = 0.702). Other subscales, including hostility (*t* = 1.464, *p* = 0.145) and fear (*t* = 1.635, *p* = 0.104), did not differ significantly between the groups.

##### Choir Context

In the context of choir participation, both groups demonstrated similar emotional responses. No significant differences were observed in the excitement (*t* = −1.174, *p* = 0.242) and motivation (*t* = −1.051, *p* = 0.295) subscales within the PAS. Similarly, negative emotions within choir in the subscale agitation (*t* = 1.239, *p* = 0.217) did not differ significantly. Although in the subscale tension, school choir singers (*M* = 1.94, *SD* = 0.741) showed slightly higher levels than extracurricular (*M* = 1.77, *SD* = 0.649), the differences were not statistically significant (*t* = 1.772, *p* = 0.078).

### 3.3. Social Support

Participants in both formal and informal choir groups reported similarly strong perceptions of social support in life. No significant differences were found in either the social connections (*t* = −0.526, *p* = 0.599) or social support (*t* = −0.516, *p* = 0.606) subscales. This indicates that choir involvement, whether in school or extracurricular contexts, contributes positively to youths’ subjective experience of supportive relationships and sense of belonging in life.

### 3.4. Mental Health

Responses regarding MHC-SF indicated no significant difference (*t* = −0.442, *p* = 0.659) in overall positive mental health between school (*M* = 2.98, *SD* = 0.887) and extracurricular (*M* = 2.93, *SD* = 0.934) choir participants. Both groups showed similar levels of emotional, psychological, and social well-being.

### 3.5. Perceived Benefits from Choral Singing

Participants in both school and extracurricular choirs reported high levels of perceived benefits from choral singing across all five CAPBES subscales. While three subscales showed no significant differences between groups, extracurricular choir members reported significantly greater social connectedness (*t* = −2.579, *p* = 0.011), as well as overall well-being (*t* = −2.419, *p* = 0.016), compared with school choir members. This suggests that youth involved in informal (extracurricular) choirs reported a stronger sense of belonging and close relationships within the choir group (e.g., “In choir, I feel like a part of a tight-knit group of friends”; “My choir mates bring very important things to my life”). Similarly, extracurricular choir singers felt more emotionally refreshed and uplifted after singing, with items highlighting feeling energized, mood improvement, relaxation, and emotional connection (e.g., “After I sing, I am in a better mood”; “After singing, I feel full of energy and vitality”; “In choir, I relax and forget my problems for a little while”). These experiences suggest informal participation may provide more restorative and emotionally fulfilling experiences, potentially due to higher intrinsic motivation, stronger group cohesion, and voluntary engagement.

### 3.6. Factors Within Choir Contributing to Well-Being

Regarding the additional question within the survey regarding choir-related factors that contribute to participants’ psychological well-being, descriptive analysis and t-test were conducted (Table 8). Among the five factors, two (F1 and F5) demonstrated a statistically significant difference between school and extracurricular choir groups.

Participants in extracurricular choirs rated F1, socializing with other choir members, significantly higher than those in school choirs (*t* = −2.732, *p* = 0.007). This suggests that community-based choirs potentially foster stronger peer relationships and a greater sense of belonging, offering a more socially enriching environment for youth. F5, choir repertoire, was rated more positively by extracurricular choir members (*t* = −2.238, *p* = 0.027), indicating that the musical program within the informal choirs may be perceived as more enjoyable, engaging, aligned with choristers’ musical preferences, or personally meaningful.

No significant differences were found between groups regarding the personal singing growth, participation in contests, or concert performances. These results suggest that while both choir settings provide similar opportunities for performance, personal growth, and skills development, extracurricular choirs may provide more social and musical satisfaction than school choirs.

### 3.7. Summary of Results

The comparative analysis of well-being, emotional experiences in life and choir context, mental health, social support, and perceived benefits from choral singing revealed both similarities and differences between school (formal) and extracurricular (informal) choir participation.

**Figure 1:**
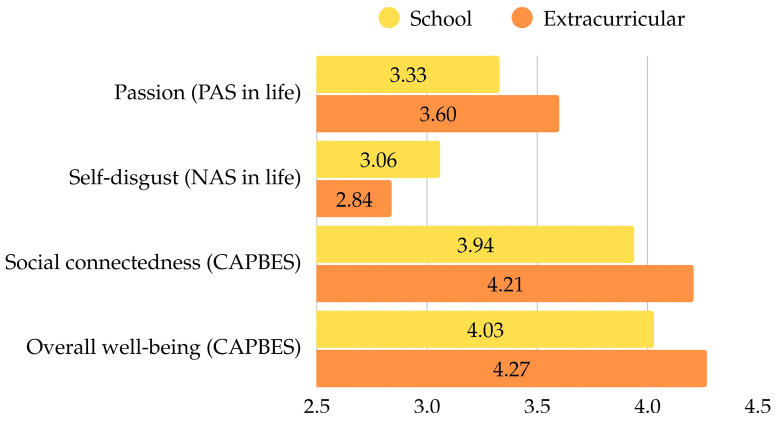
Statistically significant differences between school and extracurricular choristers across subscales and *t*-test results between groups.

No significant differences were found in general psychological well-being (WHO-5) and life satisfaction (SWLS), indicating that participation in either choir setting is associated with similar levels of overall well-being. Similarly, positive mental health (MHC-SF) did not differ between the groups. However, significant group differences emerged in emotional well-being (PANAS) in life including passion and self-disgust, as well as perceived benefits from choral singing (CAPBES), particularly in subscales social connectedness and overall well-being in choir (Figure 1).

In general life context, extracurricular choir singers reported significantly higher levels of passion, reflecting a greater sense of enthusiasm and inspiration in daily life, while school choir members experienced significantly more self-disgust, including feeling more upset, guilty, or ashamed. However, these differences (in both positive or negative affect) did not appear in the choir context. Regarding perceived benefits from choir activity, extracurricular choir members reported significantly greater levels of social connectedness and overall well-being derived from their choir experience. These findings may reflect that a more voluntary and intrinsically motivated nature of choir participation can foster deeper socio-emotional benefits, engagement, and overall well-being.

## 4. Discussion

The study explored whether the type of choir participation—school (formal) or extracurricular (informal)—is associated with differences in youth well-being, mental health, social support, and perceived benefits from choral singing. While various well-being benefits were reported across both groups, several meaningful distinctions emerged, suggesting that the hypothesis was partially confirmed.

Regarding general well-being (WHO-5), life satisfaction (SWLS), and mental health (MHC-SF), no significant differences were found between school and extracurricular choir participants. This reflects how both choir settings contribute similarly to youth overall psychological functioning and subjective life satisfaction. These outcomes align with the previous literature showing that both formal (Acquah, 2016) and informal (Barrett & Vermeulen, 2019) choir participation support positive mental health and general well-being. Although the informal choir members reported slightly higher mean scores on general well-being, highlighting the voluntary participation found in extracurricular choirs (Barrett & Vermeulen, 2019), where intrinsic motivation (personal interest, love for music) is greater, the differences were not statistically significant.

In terms of emotional experiences in life context (PANAS), significant differences emerged, particularly in the passion (PAS) and self-disgust (NAS) subscales. Extracurricular choir singers reported greater passion, characterized by enthusiasm, inspiration, and joy in life, than school choir singers. Additionally, school choir singers reported significantly higher scores on the self-disgust subscale, experiencing negative states such as shame, guilt, and distress. This may reflect that youth in formal choir settings experience more negative emotions (self-focused) and dissatisfaction with oneself. Structured school choirs often include evaluation, peer comparison, other people’s expectations, and sometimes social dynamics that can increase self-consciousness. Previous studies (Koulouriotou & Papakitsos, 2023; Parker, 2014; Petrović, 2023) have documented how peer judgment, cliques, mockery, and performance challenges in school choirs can negatively impact emotional well-being.

This highlights that youth who voluntary engage in choir singing outside of a school context tend to experience more positive emotions in daily life, compared with those participating in school-based (formal) choirs. Students involved in school choirs often participate due to external motivators such as academic obligations, school policies, grades, or teacher expectations. Consequently, emotional benefits may be more limited if the participation feels obligatory rather than chosen. However, this finding should be interpreted with caution, as the PANAS in life scale, according to the criteria, showed lower internal consistency, compared with other measures used in the study.

Interestingly, when examining emotions specifically within the choir context, both groups reported comparable levels of positive (the subscales motivation and excitement) and negative emotions (the subscales tension and agitation). This showed that during singing in a choir, regardless of settings, youth experience similar positive and negative emotions. This aligns with prior findings (Acquah, 2016; Linnemann et al., 2017) showing that choral singing itself produces immediate emotional (post-rehearsal) benefits for youth, such as relaxation, happiness, uplifted mood, and reduced stress.

Participants in both settings reported similarly high levels of perceived social support (SPS-10), with no significant differences between groups. However, while general perceptions of social support were comparable, the perceived benefits from choral singing (CAPBES) revealed important differences. Extracurricular choir singers scored significantly higher in the subscales social connectedness and overall well-being, compared with school choir singers, which are related to their choir participation. It can be inferred that voluntary engagement may foster tighter bonds and sense of community among choir members. According to self-determination theory (SDT) (Ryan & Deci, 2000), environments that support basic psychological needs (BPNs) such as autonomy, relatedness, and competence tend to increase intrinsic motivation and satisfaction, resulting in a higher sense of well-being. Informal choirs, being voluntary and self-motivated, likely fulfil these BPNs, leading to greater subjective well-being of choristers.

Moreover, the higher scores on the overall well-being subscale among extracurricular choir members, which included items related to mood improvement, energy, vitality, and relaxation, further support that informal choirs provide a more emotionally fulfilling experience. In contrast, school choir settings, while beneficial, often include a more structured program, formal obligations, and evaluations, which may diminish the sense of autonomy and intrinsic enjoyment, affecting their overall experience in the choir.

The rehearsal frequency revealed additional contextual insights. Most school choir participants rehearsed twice per week, whereas the majority of extracurricular choir members rehearsed once per week. This reflects the more structured and demanding nature of formal choirs within schools, which are often integrated as a part of the academic obligations. Prior research suggests that the number of rehearsals per week does not strongly predict well-being outcomes. Instead, long-term commitment to the activity appears to be more important for well-being. For example, Lonsdale and Day (2020) found that years of participation in an activity predicted happiness, while weekly hours did not. Similarly, the study by Jozić and Butković (2023) found that while singing frequency and group singing were associated with less negative affect, it was the perceived importance of singing that predicted higher life satisfaction and positive emotions in participants.

Regarding the specific factors within the choir that may affect youth psychological well-being, significant differences emerged in two factors, including F1, socializing with other choir members, and F5, choir repertoire. Both were rated more highly by extracurricular choir members. This could point to the stronger social connections and more engaging, personally meaningful musical content in community settings, supporting SDT’s emphasis on relatedness and autonomy (Ryan & Deci, 2000). In contrast, no differences were observed for performances, contests, or vocal development, suggesting both choir settings offer similar opportunities for musical growth, competence (Ryan & Deci, 2000), and performing. Overall, the enhanced socio-emotional benefits in informal choirs highlight the great value of voluntary and self-driven motivation for singing in a choir.

Differences in availability may explain the disparity in the number of participants. Formal (school) choirs are often integrated into the curriculum, with participation often being mandatory and linked to credits or teacher expectations. These extrinsic motivational factors may encourage students but could also limit the depth of well-being benefits when participation feels obligatory. In contrast, informal (extracurricular) choirs take place outside of school settings and attract youth who are intrinsically motivated, driven by personal interest and love for music, which likely contributes to their stronger socio-emotional benefits. However, informal choirs are far less numerous than school choirs and operate outside the formal curriculum, often requiring additional resources such as parental support, transportation, or membership fees, while time constrains and competing commitments further limit participation.

However, several limitations should be acknowledged in this study. The questionnaire included self-reported data, which may be a risk of bias and subjectivity. The self-disgust subscale demonstrated lower internal reliability; thus, the findings related to negative emotions in life should be interpreted with caution. Additionally, the absence of a longitudinal design limits our ability to assess how sustained participation in different choir settings may affect youth well-being over time.

## 5. Conclusions

The results of the study indicate that choir participation, formal or informal, contributes positively to youth well-being, positive mental health, social support, and personal growth, including musical development and performance opportunities. Notably, youth involved in extracurricular choirs experience greater social connectedness, vitality, and stronger socio-emotional benefits related to choir participation, such as social interaction with other peers in choir and enjoying the choir repertoire. The higher levels of positive affect and lower levels of negative affect in life reported by extracurricular choir members emphasize the value of informal choir settings in supporting youth emotional well-being.

The disparity in participant numbers between school and extracurricular choir groups both highlight a methodological limitation and reflect a broader issue—the limited access to community-based choir opportunities for youth. While school choirs are more widely established and often integrated into educational systems, the smaller number of respondents from extracurricular choirs suggests that such programs are less common. Given the socio-emotional well-being benefits of informal choir participation found in this study, creating and expanding community choir initiatives could help youth thrive by providing a space for emotional relief, social connection, and overall well-being through meaningful musical engagement. From a policy perspective, these findings suggest that school choir programs could be enhanced by adopting practices more characteristic of informal (extracurricular) choirs. Providing greater student autonomy in repertoire selection; reducing the emphasis on evaluation, grades, or other requirements; and framing participation as a voluntary, rather than mandatory, activity may increase intrinsic motivation and associated well-being benefits. Educational policies that prioritize autonomy, social connectedness, and meaningful musical experiences could help school choirs mirror the socio-emotional benefits observed in informal choir settings.

## Figures and Tables

**Table 1:** Previous studies involving youth choir singers (school/extracurricular).

Choir Settings	Study and Year	Sample	Well-Being Benefits
Formal	Acquah (2016)	University (emerging adults)	Emotional (reduced stress, improved mood) Psychological (self-confidence, concentration, sense of accomplishment, responsibility, discipline, motivation for academic success, social skills) Social (increased interaction, connectedness, sense of belonging) Physical (posture and breathing)
	Stojanović and Milošević (2019)	School (adolescents)	
	Koulouriotou and Papakitsos (2023)		
Informal	Barrett and Vermeulen (2019)	University (emerging adults)	Emotional (more positive and less negative emotions, reduced stress) Social (cultural connections, interaction, inclusion, sense of belonging, social support, teamwork, less loneliness) Psychological (time management skills, discipline, concentration, self-confidence, creativity, less shyness) Physical health
	Linnemann et al. (2017)		
	Petrović (2023)	Extracurricular (adolescents)	
Formal and informal	Glew et al. (2020)	School/extracurricular (adolescents)	Psychological (self-confidence, positive self-perception, leadership skills, future goals, sense of pride, external validation) Emotional (mood regulation) Social (inclusion, social connectedness, cooperation, social identity)
	Parker (2014)		

**Table 2:** Participant demographics.

Characteristics	*N*	%
Sex		
Male	50	18.2%
Female	223	81.4%
Other	1	0.4%
Age group		
15–19	198	72.3%
20–24	76	27.7%
Country		
Serbia	95	34.7%
Slovenia	179	65.3%
Choir settings		
School	197	71.9%
Extracurricular	77	28.1%
Choir type		
Mixed	147	53.6%
Female	108	39.4%
Male	19	6.9%

**Table 3:** School and extracurricular choir distribution across groups.

Youth Age Group	Choir Type	*N*	% of Total	% Within the Group
Adolescents (15–19)	School	157	57.3%	79.7%
	Extracurricular	41	15%	20.8%
Emerging adults (20–24)	School	40	14.6%	52.6%
	Extracurricular	36	13.1%	46.8%

**Table 4:** Reliability analysis.

Scale	Cronbach’s α	CFI	TLI	RMSEA
WHO-5 (whole scale)	0.816	0.942	0.885	0.144
SWLS (whole scale)	0.798	0.954	0.908	0.125
PAS in life (factor refinement)	0.678	0.839	0.773	0.088
NAS in life (factor refinement)	0.782	0.772	0.680	0.132
PAS in choir (factor refinement)	0.876	0.959	0.946	0.069
NAS in choir (one item excluded)	0.847	0.910	0.842	0.122
CAPBES (factor refinement)	0.926	0.922	0.909	0.072
MHC-SF (factor refinement)	0.908	0.865	0.841	0.106
SPS-10 (factor refinement)	0.901	0.932	0.907	0.101

**Table 5:** Rehearsal frequency.

Rehearsals per Week	Choir Settings	*n*	% Within Group	Total %
1	School	197	46 (23.3%)	101 (37.1%)
	Extracurricular	77	55 (71.4%)	
1–2	School	197	5 (2.6%)	8 (2.9%)
	Extracurricular	77	3 (3.9%)	
2	School	197	109 (55.3%)	122 (44.9%)
	Extracurricular	77	13 (16.9%)	
2–3	School	197	6 (3%)	8 (2.9%)
	Extracurricular	77	2 (2.6%)	
3	School	197	27 (13.7%)	29 (10.7%)
	Extracurricular	77	2 (2.6%)	
3–4	School	197	0 (0.0%)	1 (0.4%)
	Extracurricular	77	1 (1.3%)	
4	School	197	3 (1.5%)	3 (1.1%)
	Extracurricular	77	0 (0.0%)	

Note. Percentages in % within group refer to each choir settings (school/extracurricular). Percentages in Total % refer to the whole sample (n = 274).

**Table 6:** Descriptive statistics for all tests—Choir type differences.

Instruments	Factors	Group	*N*	*M*	*SD*	SE
SWLS	One factor	School	197	4.55	1.094	0.0780
		Extracurricular	77	4.49	0.969	0.1105
WHO-5	One factor	School	197	2.69	0.886	0.0631
		Extracurricular	77	2.90	0.847	0.0965
PAS in life	Energy	School	197	3.17	0.797	0.0568
		Extracurricular	77	3.16	0.859	0.0979
	Passion	School	197	3.33	0.888	0.0633
		Extracurricular	77	3.60	0.855	0.0974
	Engagement	School	197	3.61	0.574	0.0409
		Extracurricular	77	3.63	0.649	0.0740
NAS in life	Hostility	School	197	2.50	0.820	0.0584
		Extracurricular	77	2.34	0.845	0.0963
	Self-disgust	School	197	3.06	0.727	0.0518
		Extracurricular	77	2.84	0.702	0.0800
	Fear	School	197	3.14	0.819	0.0583
		Extracurricular	77	2.98	0.722	0.0823
PAS in choir	Excitement	School	197	3.76	0.784	0.0558
		Extracurricular	77	3.88	0.729	0.0831
	Motivation	School	197	3.89	0.766	0.0546
		Extracurricular	77	3.98	0.627	0.0714
NAS in choir	Agitation	School	197	1.57	0.577	0.0411
		Extracurricular	77	1.48	0.515	0.0586
	Tension	School	197	1.94	0.741	0.0528
		Extracurricular	77	1.77	0.649	0.0740
CAPBES	Ability	School	197	4.18	0.804	0.0573
		Extracurricular	77	4.25	0.729	0.0831
	Optimism	School	197	3.27	0.941	0.0670
		Extracurricular	77	3.40	0.946	0.1078
	Social connectedness	School	197	3.94	0.885	0.0631
		Extracurricular	77	4.21	0.728	0.0830
	Overall wellbeing	School	197	4.03	0.912	0.0650
		Extracurricular	77	4.27	0.657	0.0748
	Accomplishment	School	197	4.43	0.739	0.0526
		Extracurricular	77	4.50	0.641	0.0730
MHC-SF	One factor	School	197	2.98	0.887	0.0632
		Extracurricular	77	2.93	0.934	0.1065
SPS-10	Social support	School	197	3.58	0.517	0.0368
		Extracurricular	77	3.61	0.458	0.0522
	Social connections	School	197	3.48	0.468	0.0334
		Extracurricular	77	3.51	0.446	0.0508

**Table 7:** Independent t-test for all tests—Choir type differences.

Instruments	Factors	*t*	*Df*	*p*	*M* Difference	SE Difference
SWLS	One factor	0.488	156	0.626	0.0660	0.1352
WHO-5	One factor	−1.815	145	0.072	−0.2094	0.1153
PAS in life	Energy	0.148	130	0.883	0.0167	0.1132
	Passion	−2.310 *	144	0.022	−0.2684	0.1162
	Engagement	−0.231	125	0.817	−0.0196	0.0846
NAS in life	Hostility	1.464	135	0.145	0.1649	0.1126
	Self-disgust	2.292 *	143	0.023	0.2184	0.0953
	Fear	1.635	156	0.104	0.1649	0.1009
PAS in choir	Excitement	−1.174	148	0.242	−0.1175	0.1001
	Motivation	−1.051	168	0.295	−0.0945	0.0899
NAS in choir	Agitation	1.239	155	0.217	0.0888	0.0716
	Tension	1.772	157	0.078	0.1610	0.0909
CAPBES	Ability	−0.711	152	0.478	−0.0718	0.1010
	Optimism	−0.991	138	0.323	−0.1258	0.1269
	Social connectedness	−2.579 *	167	0.011	−0.2687	0.1042
	Overall well-being	−2.419 *	192	0.016	−0.2397	0.0991
	Accomplishment	−0.840	159	0.402	−0.0756	0.0900
MHC-SF	One factor	0.442	133	0.659	0.0548	0.1238
SPS-10	Social support	−0.516	156	0.606	−0.0330	0.0639
	Social connections	−0.526	145	0.599	−0.0320	0.0608

Note. * for *p* < 0.05.

**Table 8:** Factors within choir enhancing well-being.

Factors	Choir Settings	*M*	*SD*	*t*	*p*
F1 Socializing with other choir members	School	3.87	0.938	−2.732 *	0.007
	Extracurricular	4.17	0.768		
F2 Achieving personal singing potential	School	3.85	0.939	−0.141	0.888
	Extracurricular	3.87	0.908		
F3 Participating in contests	School	3.25	1.16	1.400	0.164
	Extracurricular	3.01	1.32		
F4 Performing concerts	School	3.96	0.992	0.754	0.452
	Extracurricular	3.86	1.08		
F5 Choir repertoire	School	3.64	1.01	−2.238 *	0.027
	Extracurricular	3.91	0.846		

Note. * for *p* < 0.05.

## Data Availability

The original contributions presented in this study are included in the article. Further inquiries can be directed to the corresponding author.

## Abbreviations

The following abbreviations are used in this manuscript

BPNs	Basic psychological needs
CAPBES	Choral Activity Perceived Benefits Scale
CFA	Confirmatory factor analysis
CFI	Comparative fit index
EFA	Exploratory factor analysis
MHC-SF	Mental Health Continuum Short Form
NAS	Negative Affect Schedule
PANAS	Positive and Negative Affect Schedule
PAS	Positive Affect Schedule
RMSEA	Root mean square error of approximation
SEM	Structural equation modeling
SDT	Self-determination theory
SPS	Social Provision Scale
SWLS	Satisfaction with Life Scale
TLI	Tucker–Lewis index
WHO	World Health Organization
